# Supplementary material for: A simple and robust method for automating analysis of naïve and regenerating peripheral nerves
Source: PLoS One. 2021 Jul 7;16(7):e0248323. doi: 10.1371/journal.pone.0248323 (PMC8263263; doi:10.1371/journal.pone.0248323)
Supplement: Attachment — Submitted filename: 2021.05.26.response to reviewers.docx [file pone.0248323.s001.docx]

Alison Wong, MD MSE FRCSC

Department of Plastic and Reconstructive Surgery

Johns Hopkins School of Medicine – Ross Research Building

720 Rutland Ave., Room 749A

Baltimore, MD, USA 21205

May 26, 2021

PLOS ONE

Re: Response to Reviewers

Thank you for the opportunity to submit our revised manuscript, “A simple and robust method for automating analysis of naïve and regenerating peripheral nerves.” We believe that the comments and suggestions have significantly improved the manuscript and hope that you find the changes satisfactory. Below are the itemized responses to the reviewers along with the corresponding line numbers where applicable.

**Did the authors note any differences between embedding protocols? Resin vs. paraffin. This would be interesting to know as this has implications on time commitment to sample preparation.**

Our lab has been using both embedding protocols due to the increased accessibility and decreased costs of paraffin embedding, we have also found the paraffin protocol to be somewhat faster. A statement to this effect has been added to the methods section (Line 152).

**Have the authors attempted to manually correct the ADS data to determine if this would further improve the accuracy? As ADS much faster than manual segmentation, it may be beneficial to include this step if this is not a time consuming process.**

We tried to only do minimal corrections. More in-depth corrections likely would have improved the accuracy of axon counts, but we do not think that it would have changed the differences seen in the measurements of axon diameter or myelin thickness. This was mentioned in our methods but has also been added to our discussion (Line 329).

**The discussion would be improved if this methodology is compared with other published reports. This is not to say the authors should implement other methodologies but compare other authors result with their own.**

More details around the methodology used in other reports has been added. Briefly it was combined with the rationale behind using ADS in the introduction (Line 103), and more thoroughly methodology is now compared in the discussion (Line 435).

**Can the authors comment on the efficacy of counting non-myelinated axons. This is the bigger challenge and is important when considering sensory nerve recovery.**

We completely agree that there is an important role of the analysis of non-myelinated axons for sensory nerves. For this study, we focused on light microscopy, where the common methods of tissue preparation and staining focus on staining the myelin itself. Sensory nerves typically are analyzed using electron microscopy. ADS was developed in electron micrographs and likely could be used for the analysis for unmyelinated axons, but this has not yet been tested. We have added this to the conclusion as a future area of interest (Line 468).

**The quality of the figures is quite low; please improve the resolution of the figures showing the data (Figures 1/2 appear fine).**

We are not sure why this is the case and have now verified them using the PACE figure application so the resolution should not be a problem.

**Please, consider moving your motivation for using ADS to the introduction, rather than the discussion, so the reader understands up front why you are studying this software in particular.**

Thank you for the suggestion, this has been done (Line 103 and 110).

**Please, consider introduction of the g-ratio definition early.**

The definition of g-ratio is present in the description of the methodology used in the manual analysis (Line 214), the definition has now also been added to the “Measurements” section of the results for increased clarity (Line 325).

**Line 77: Period missing.**

This has been added.

**Line 175: Can you explain the ‘minferet’ command for users not familiar with imagej?**

This has been added (Line 215).

**Line 196: Does the GIMP software automatically label these images? Any reason why the software was not compared using this software?**

I am not familiar with using GIMP for labeling images. I know that in GIMP or Photoshop images can be overlaid to visually assess differences in the images. The larger issue is that with Fiji/ImageJ, the thresholded images are not automatically saved, so we could not compare the graphic outputs of each software program (ImageJ vs. ADS).

**Line 362 a citation has not been included.**

This sentence was referring back to our results, and therefore a citation was not included. The sentence itself has been changed to make this more clear. “*We found that ADS was able to appropriately identify differences in axon diameter, myelin thickness and g-ratio between naïve and regenerating nerves.*” (now found on line 414)

**Line 363: Ref not inserted**

Line 363 is referring to findings, perhaps the reviewer meant line 373? An appropriate citation has been added to that statement to provide support (Line 453).

**Please, provide in the INTRODUCTION examples of recent works on peripheral nerve regeneration (see, for example doi: 10.1088/1758-5090/aaaf50).**

A brief overview of some of the advances in peripheral nerve regeneration have been added to the introduction (Line 72).

Thank you for your consideration,

Alison Wong, MD, MSE, FRCS(C)
